# The reduction of astrocytes and brain volume loss in anorexia nervosa—the impact of starvation and refeeding in a rodent model

**DOI:** 10.1038/s41398-019-0493-7

**Published:** 2019-06-04

**Authors:** Linda Frintrop, Stefanie Trinh, Johanna Liesbrock, Christina Leunissen, Julia Kempermann, Serhat Etdöger, Martien J. Kas, René Tolba, Nicole Heussen, Joseph Neulen, Kerstin Konrad, Vera Päfgen, Fabian Kiessling, Beate Herpertz-Dahlmann, Cordian Beyer, Jochen Seitz

**Affiliations:** 1 Institute of Neuroanatomy, RWTH Aachen University, 52074 Aachen, Germany; 2 Department of Child and Adolescent Psychiatry, Psychosomatics and Psychotherapy, University Hospital, RWTH Aachen University, 52074 Aachen, Germany; 3 Department of Translational Neuroscience, Brain Center Rudolf Magnus, University Medical Center Utrecht, 3584 CG Utrecht, The Netherlands; 4 Groningen Institute for Evolutionary Life Sciences, University of Groningen, Groningen, The Netherlands; 5 Institute for Laboratory Animal Science and Experimental Surgery, University Hospital Aachen, RWTH Aachen University, 52074 Aachen, Germany; 6 Department of Medical Statistics, University Hospital Aachen, RWTH Aachen University, 52074 Aachen, Germany; 7 Center of Biostatistics and Epidemiology, Sigmund Freud University, 1030 Vienna, Austria; 8 Department of Gynecological Endocrinology and Reproductive Medicine, University Hospital, RWTH Aachen University, 52074 Aachen, Germany; 9 Experimental Molecular Imaging, RWTH Aachen University, 52074 Aachen, Germany

**Keywords:** Psychiatric disorders, Molecular neuroscience

## Abstract

Anorexia nervosa (AN) is an often chronic, difficult to treat illness that leads to brain volume reductions in gray and white matter. The underlying pathophysiology is poorly understood, despite its potential importance in explaining the neuropsychological deficits and clinical symptoms associated with the illness. We used the activity-based anorexia model (ABA), which includes food reduction and running wheel access in female rats to study brain changes after starvation and refeeding. Longitudinal animal MRI and post-mortem brain sections confirmed a reduction in the mean brain volumes of ABA animals compared to controls. In addition, the mean number of astrocytes was reduced by over 50% in the cerebral cortex and corpus callosum, while the mean number of neurons was unchanged. Furthermore, mean astrocytic GFAP mRNA expression was similarly reduced in the ABA animals, as was the mean cell proliferation rate, whereas the mean apoptosis rate did not increase. After refeeding, the starvation-induced effects were almost completely reversed. The observation of the astrocyte reduction in our AN animal model is an important new finding that could help explain starvation-induced neuropsychological changes in patients with AN. Astrocyte-targeted research and interventions could become a new focus for both AN research and therapy.

## Introduction

Anorexia nervosa (AN) is the third most common chronic disease in adolescents, with the highest mortality rate of any mental illness^1^. It is characterized by a combination of insufficient energy intake and often increased physical activity that results in severe weight loss^2,3^. Psychotherapy shows low effectiveness during the starved state. Medical intervention is very limited, and there is no approved pharmacological treatment available for patients with AN^4^. The relapse rate in AN is approximately 25–50% in the first year^5^, and the pathophysiology of AN is not well understood^6^. As a consequence of starvation, severe volume reductions in gray and white brain matter have been found in patients with AN^7^. In two meta-analyses by our group that included 473 patients with AN, a reduction of 4.6% in gray matter and 2.7% in white matter was found in patients with AN compared to healthy controls^7,8^. These brain matter reductions were associated with neuropsychological deficits, such as cognitive impairments in learning and visuospatial memory^8–12^. The gray matter loss was correlated with an increased desire for thinness in AN patients^13^ and was linked to a worse clinical outcome after one year^12^. After long-term body weight restoration, the brain volume changes appear to be reversible^7,14^. However, chronically underweight patients seem to have progressive brain changes^15^. The underlying pathomechanism of this brain volume reduction in AN is largely unknown. King *et al*. analyzed several measures of serum and urine osmolarity and thereby showed that a volume reduction solely based on fluid shifts appears unlikely (also see Vogel et al.^16^, King et al., 2018^17^). Furthermore, no alteration of the glial markers GFAP and S100b or the neuronal marker NSE could be found in the serum of the patients with AN, making it necessary to perform direct brain analyses^18,19^. Systematic post-mortem data from human brain studies are rarely available, as only two human post-mortem case studies are known that analyzed the brains of 3 patients with AN. These studies suggested the cellular degeneration of neurons and an altered spine morphology^20,21^. Translational animal research could help to unravel the cellular mechanism of these observed volumetric brain changes.

The best-established animal model that mimics the core symptoms of AN, such as weight loss, hyperactivity, and amenorrhea, is the so-called activity-based anorexia (ABA) paradigm^22^, which combines restricted food availability with access to a running wheel. This combination results in starvation and voluntary hyperactivity in ABA animals; this seemingly contradictory activity can possibly be explained as food seeking behavior and is driven by lower leptin levels^22,23^. We established a modified version of the original ABA model by giving a limited amount of food instead of a limited time for feeding with the aim of preventing mortality in the animals^24,25^.

In a proof-of-concept study, our group showed that brain volume reductions in the corpus callosum and the cerebral cortex in the rat model parallel those of human studies^26^. The pilot study found a reduction in glial fibrillary acidic protein (GFAP)-positive astrocytes in the cortex and corpus callosum, potentially underlying this brain volume reduction. Furthermore, there was no change in neuronal cell number. This finding could hint at a completely overlooked mechanism in the pathomechanism of volume reduction in AN. Astrocytes play an important role in providing neurons with nutrients and modulating neurotransmitter reuptake and synaptic plasticity^27^. There is increasing evidence for their involvement in complex phenomena such as sleep homeostasis as well as cognitive deficits associated with sleep loss and depression, potentially due to synaptic dysfunction^28,29^. Astrocyte reductions have also been shown by Reyes-Haro *et al*^22^. in a dehydration-induced rat model of AN and in patients with depression^28^. The deletion of frontal astrocytes was even found to causally induce depressive symptoms in rats^23^. Furthermore, Barbarich-Marsteller et al.^30^ showed that the neogenesis of predominantly glial cells in the hippocampus and corpus callosum was reduced in ABA rats^30^. Thus, astrocyte loss and reduced cell neogenesis could play an important role in influencing neuronal function and could potentially help explain some of the neuropsychological deficits, such as rigidity, memory deficits and impaired learning, in AN. These symptoms render the psychotherapeutic process difficult, especially during acute starvation.

The principal aim of our study was to clarify the underlying mechanism of brain volume loss in AN. Therefore, we investigated the reduction of astrocytes using immunohistochemical staining and mRNA expression analysis in a large group of ABA rats compared to control animals. We further analyzed whether ad libitum refeeding for 20 days resulting in bodyweight restoration could reverse the starvation-induced brain volume and astrocyte reduction in ABA rats. To verify the translational importance of these findings, we analyzed brain volume changes using longitudinal MRI measurements. Finally, immunohistochemical staining was applied to visualize proliferating and apoptotic cells to gain insight into the origin of the astrocyte reduction.

## Methods and materials

### Animals

Experiments were conducted on 47 4-week-old female Wistar rats (Crl:WI, Charles River, Sulzfeld, Germany), with an average body weight of 87.93 g (SD 9.03). Female rats were used because of the higher prevalence of AN in female patients than male patients and because of the possibility of using amenorrhea as a quality control symptom indicating an adequate level of starvation. The animals were individually housed in Type IV cages (Polysulfone, Tecniplast GmbH, Hohenpeißenberg, Germany) under a 12-h light/dark cycle, and all rats had 24 h/day running wheel access. The animal room was maintained at a standard temperature (21 ± 1 °C). The facility was specific pathogen-free according to the FELASA Guidelines and certified according to DIN ISO 9001:2008. The animal procedure was permitted by the Governmental Animal Care and Use Committee LANUV North Rhine Westphalia (Landesamt für Umwelt, Natur, und Verbraucherschutz, Recklinghausen, Germany). All tests were performed in accordance with the German legislation governing animal studies following the Guide for the Care and Use of Laboratory Animals (NIH publication, 8th edition, 2011) and the 2010/63/EU Directive on the protection of animals used for scientific purposes (Official Journal of the European Union, 2010).

### Study design

The modified ABA model was established in our laboratory, and a detailed description is given in^24^. In brief, all rats had ten days to acclimatize to their individual cages and had ad libitum (ad lib, unrestricted) access to food and water during this period. The animals were randomly picked and assigned to the control and ABA groups. Afterwards, all ABA animals received 40% of their prior daily food intake (calculated with the feeding amount of the acclimatization phase) until a 25% body weight loss occurred. This acute starvation phase was followed by a two-week long chronic starvation period in which we increased the food intake to 60–80% of the original daily food intake, keeping the body weight stable at −25%. If the body weight differed by more than 2.5% compared to the target weight, the food was increased or decreased in increments of 5%. Half of the animals were sacrificed after this chronic starvation period (ABA: *n* = 12), while the other half were refed ad lib for 20 days to model weight rehabilitation (ABA_R: *n* = 11). The control groups were housed under the same conditions but were fed ad lib during the whole experiment (controls: *n* = 12, controls_R: *n* = 12). Sample size of 12 per group was chosen to match our previous study. Body weight, food intake and running wheel activity (RWA) were measured daily. Due to the study design the carers were not blinded to group allocation of the animals, however, all outcome measurements were conducted blinded.

### MRI-based brain volume measurement

MRI experiments were conducted in the refed group (ABA_R + controls_R) after habituation, after chronic starvation and after refeeding on a 1 T Bruker ICON horizontal bore that was a dedicated animal scanner (Bruker Biospin, Ettlingen, Germany). After the induction of anesthesia with 5% isoflurane, the rats were placed in an MRI-compatible cradle, and each head was fixed in place with tape. During the entire scan, animals were anesthetized with 2% isoflurane (Forene, 100%, v/v, B506, Wiesbaden, Abbott) in oxygen-enriched air. T2-weighted imaging (T2 W, FLASH sequence, field of view: 50 cm, slice thickness = 1 mm, TE = 12 ms, TR = 645 ms) was chosen for the anatomical details of the images. Using the Imalytics Preclinical 2.0 software, the total brain volume of each rat was manually segmented by one independent analyst blinded to the experimental groups, excluding the bulbus olfactorius and the brainstem but including the cerebellum^31^.

### Histological brain volume measurement

The rats were transcardially perfused with artificial cerebrospinal fluid solution, and the brains were separated into two hemispheres at the midsagittal line. We used the left hemisphere for mRNA analysis and the right hemispheres for immunohistochemistry and volume analysis. Therefore, the right halves were post-fixed with a 3.7% paraformaldehyde solution and cryo-protected by immersion with 10 and 30% sucrose. For embedding, we used an optimal cutting temperature medium. Using a cryostat (Leica CM 3050S, Nussloch, Germany), the whole right brain hemispheres were cut frontally in a series of 100 µm sections. To analyze the volumes, hematoxylin-eosin staining was performed. After digitalization, the areas of the respective regions of interest were measured by tracing with ImageJ software (1.48 v, Wayne Rasband, National Institutes of Health, USA). We then used the Cavalieri method by multiplying the individual areas by the slice thickness and adding the results to calculate the volumes of interest. The results of two independent observers were averaged, and the analysis was performed as previously described^26^ (cerebral cortex analysis from Bregma 5.2 to 9.8, corpus callosum 3.7 to 8.0).

### Immunohistochemistry

At Bregma −2.30, we made a series of 20 µm sections. The histochemical stainings were performed using standard procedures, as previously reported^26^. The following antibodies were used: goat GFAP (astroglia, 1:750, catalog number: sc-6170, Santa Cruz, USA), rabbit anti-microtubule-associated protein 2 (Map2, neurons, 1:1.500, catalog number: 8707, Cell Signaling), rabbit anti-Ki67 (proliferation marker, 1:3.000, catalog number: ab16667, Abcam) and rabbit anti-cleaved caspase 3 antibody (apoptosis marker, 1:800, catalog number: mAB#9664, Cell Signaling).

### Quantification of immunohistochemical parameters

Two immunohistochemically stained samples per analysis were digitalized and analyzed with ImageJ 3 software (1.48 v, Wayne Rasband, National Institutes of Health, USA) by two independent, blinded observers, and the results were averaged. The observers counted all GFAP-, Ki67- and caspase-3-positive cells containing a visible nucleus and expressed this value as cells/mm^2^. Then, they determined the area covered by GFAP staining to quantify the GFAP signaling in the area as %. Within the cerebral cortex analysis, three different regions (retrosplenial granular cortex, primary motor cortex and primary somatosensory cortex) were averaged. Similarly, the corpus callosum was measured in three regions (next to the midline, under the subcingulum and laterally). The regions of interest for quantifying the different cellular parameters are illustrated in Supplementary Fig. 3. Ki67 staining analysis was performed for the midline region. Like in our previous study, we excluded slices, if the area containing the ROI was damaged.

### Reverse transcription (RT) and real-time polymerase chain reaction (rtPCR)

The mRNA of the cerebral cortex and the corpus callosum (tissue from the left brain hemispheres) were isolated with peqGold RNA Trifast (Peqlab, Germany), as previously described^26^. All samples were reverse transcribed in complementary DNA, and their relative expression was analyzed by calculating the ratio between the gene of interest and the reference gene, cyclophilin A (cycA; primer sequences: CycA sense: 5′-GGCAAATGCTGGACCAAACAC, CycA antisense: 5′-TTAGAGTTGTCCACAGTCGGG AGATG; GFAP sense: 5′-AGAAAACCGCATCACCATT, antisense: 5′-GCACACCTCACATCACATCC). Alterations in the levels of genes of interest were graphically shown by the fold change relative to the control group of the same region (controls set to 100%).

### Statistical analysis

The repeatedly measured MRI brain volumes in ABA_R and control_R rodents were analyzed using a linear mixed effects model calculation with random intercept and unstructured covariance matrix to allow for baseline differences in brain volume between animals. One ABA brain volume could not be analyzed because of poor scan quality. Comparisons between ABA_R and control_R animals after starvation and after refeeding were evaluated by corresponding linear contrasts. Model assumptions and model fit were checked by visual inspection of the residuals and the measures of influence diagnostic. Missing values were taken into account by a likelihood-based approach within the framework of mixed linear models with the assumptions that missing values occur at random. For all comparisons, the significance level was set to 5%. Due to the explorative nature of this study, no adjustment was made to the significance level. The results are reported as the means and standard deviations (±SD). Two-sided p-values were accompanied by values of the test statistic (t) and degrees of freedom (t(df)). In addition, 95% confidence intervals (CI) for the difference in mean brain volume between ABA and control rats after starvation and after refeeding were provided. These analyses were performed with SAS version 9.4 (PROC MIXED; SAS Institute Inc., NC, USA).

The cellular outcome data were described as the means and corresponding standard deviations in each starvation and refeeding subgroups of the ABA and control animals. The primary outcome was the astrocyte cell number, and secondary outcomes were cerebral cortex and corpus callosum volume, astrocyte cell size and cell surface, numbers of Map2, Ki67 and Casp3-positive cells as well as mRNA expression levels. Two-sided t-tests with significance levels of 5% and corresponding degrees of freedom (t(df)), values of the test statistic (t) and effect sizes (Cohen’s d) were performed to compare the ABA and control animals. No deviation from the normal distribution was detected by the Kolmogorov-Smirnov test. These statistics were performed with SPSS version 20 for Windows (IBM, Chicago, USA).

## Results

**Fig. 1:**
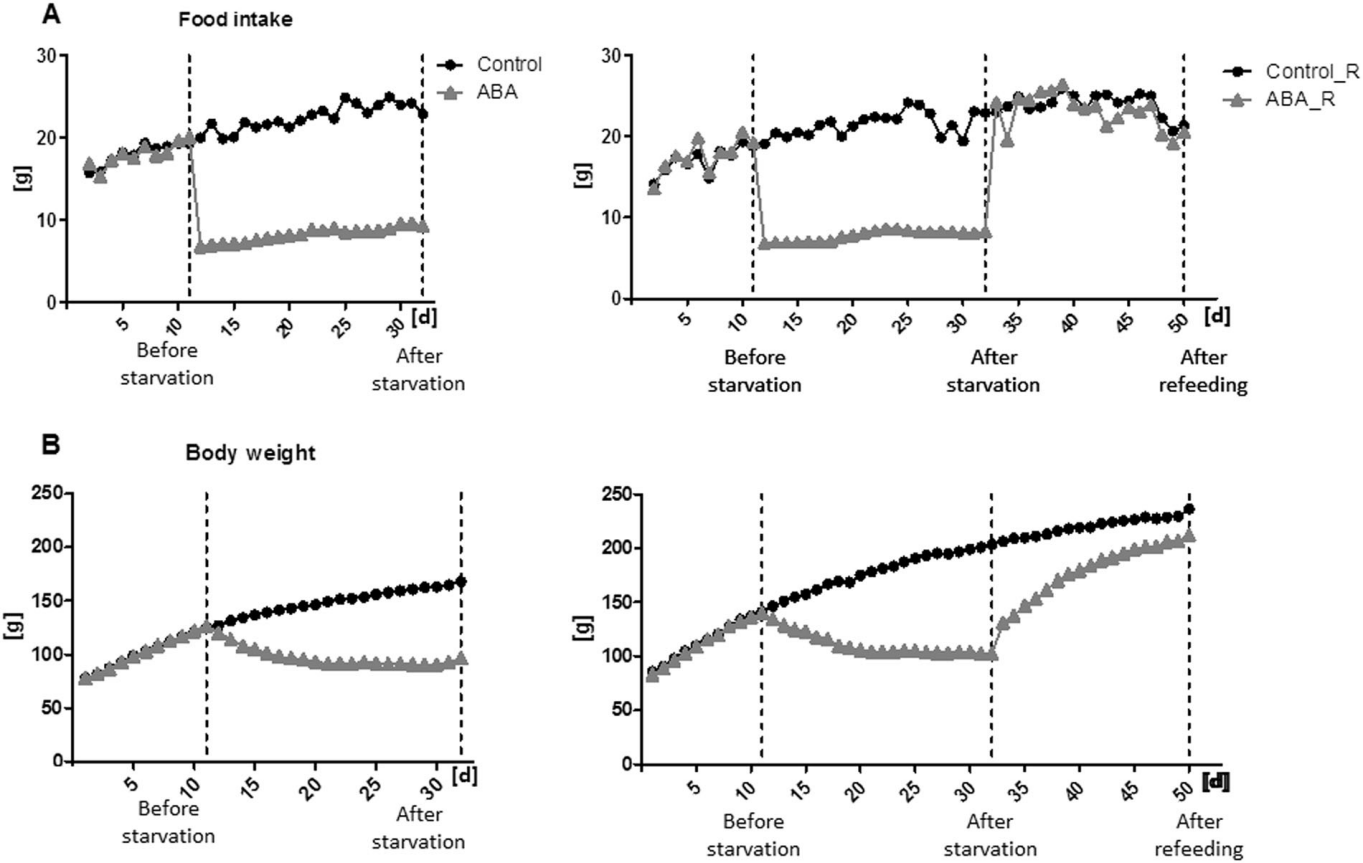
Mean food intake (**a**) and mean body weight (**b**) during the starvation of ABA and control animals (left) and during the starvation and refeeding of ABA_R and control_R animals (right)

The mean food intake and mean body weight of both cohorts are depicted in Fig. 1 (RWA in Supplementary Fig. 1).

**Fig. 2:**
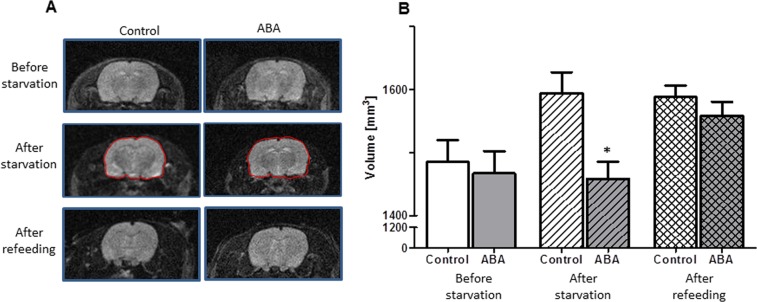
Consequences of refeeding on the total brain volume as determined by MRI analysis. The figure depicts exemplary MRI images (**a**) and the mean total brain volume and corresponding standard deviation of ABA and control animals at three different time points (**b**): before starvation, after starvation and after refeeding. Analysis of MRI brain volume in the ABA and control animals was performed using a linear mixed effects model with a random intercept and unstructured covariance. Pairwise comparisons were evaluated using the corresponding linear contrasts. *p ≤ 0.05

The MRI whole-brain volume analysis showed that, on average, the brains of the ABA animals were significantly smaller than those of the controls after starvation (t(df) = 40.7, t = −2.54, p = 0.02, Fig. 2, Supplementary Table 1). This difference was not observed after refeeding (t(df) = 40.7, t = −0.70, p = 0.49, Fig. 2).

**Fig. 3:**
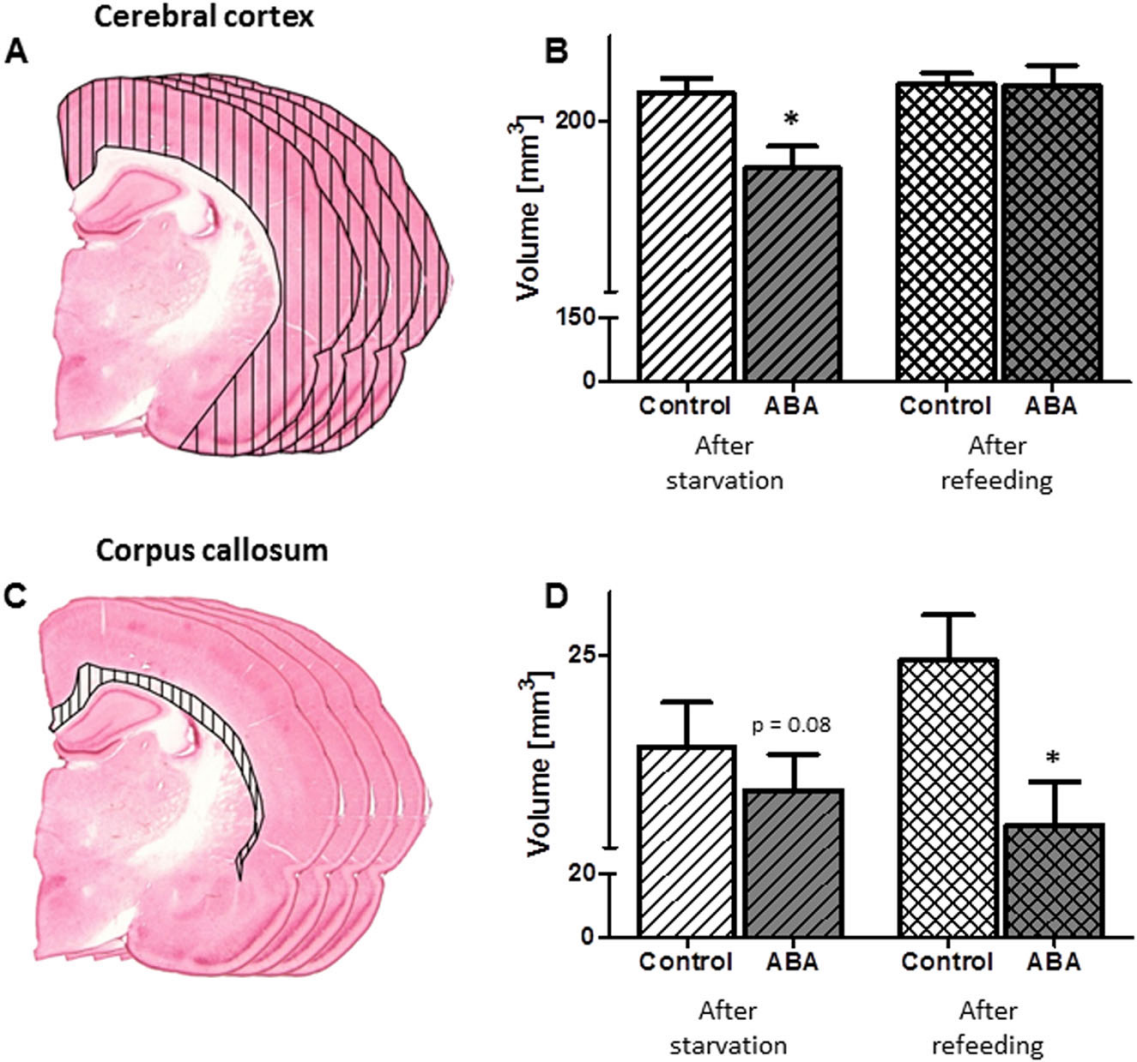
Mean total brain volume and corresponding standard deviation. Effects of starvation and refeeding on the cerebral cortex (**a**, **b**) and corpus callosum volume (**c**, **d**) after starvation and refeeding, as analyzed with serial slide measurement. *p ≤ 0.05, two-sided Student’s *t*-test

The quality of the histological slides that were used for the histological volume detection was adequate for the analysis of the cortex (controls: *n* = 10; ABA: *n* = 11, controls_R: *n* = 12, and ABA_R: *n* = 10) and the corpus callosum (controls: *n* = 7; ABA: *n* = 11, controls_R: *n* = 12, and ABA_R: *n* = 11, Supplementary Table 2). The mean cerebral cortex volume was significantly decreased by 9% after starvation in the ABA rats compared to the control group (t(df) = 19, t = 2.85, Cohen’s d = 1.24, *p* = 0.01, Fig. 3). The mean corpus callosum volume of the ABA group was 6% smaller than that of the control group (t(df) = 16, t = 1.89, Cohen’s d = 0.91, *p* = 0.08). After refeeding, the difference in the cerebral cortex volumes between the groups showed no statistically significant difference (*p* = 0.57); however, the corpus callosum volume was significantly reduced by 10% in the ABA_R animals compared to the controls (t(df) = 21, t = 2.42, Cohen’s d = 1.01, *p* = 0.02).

**Fig. 4:**
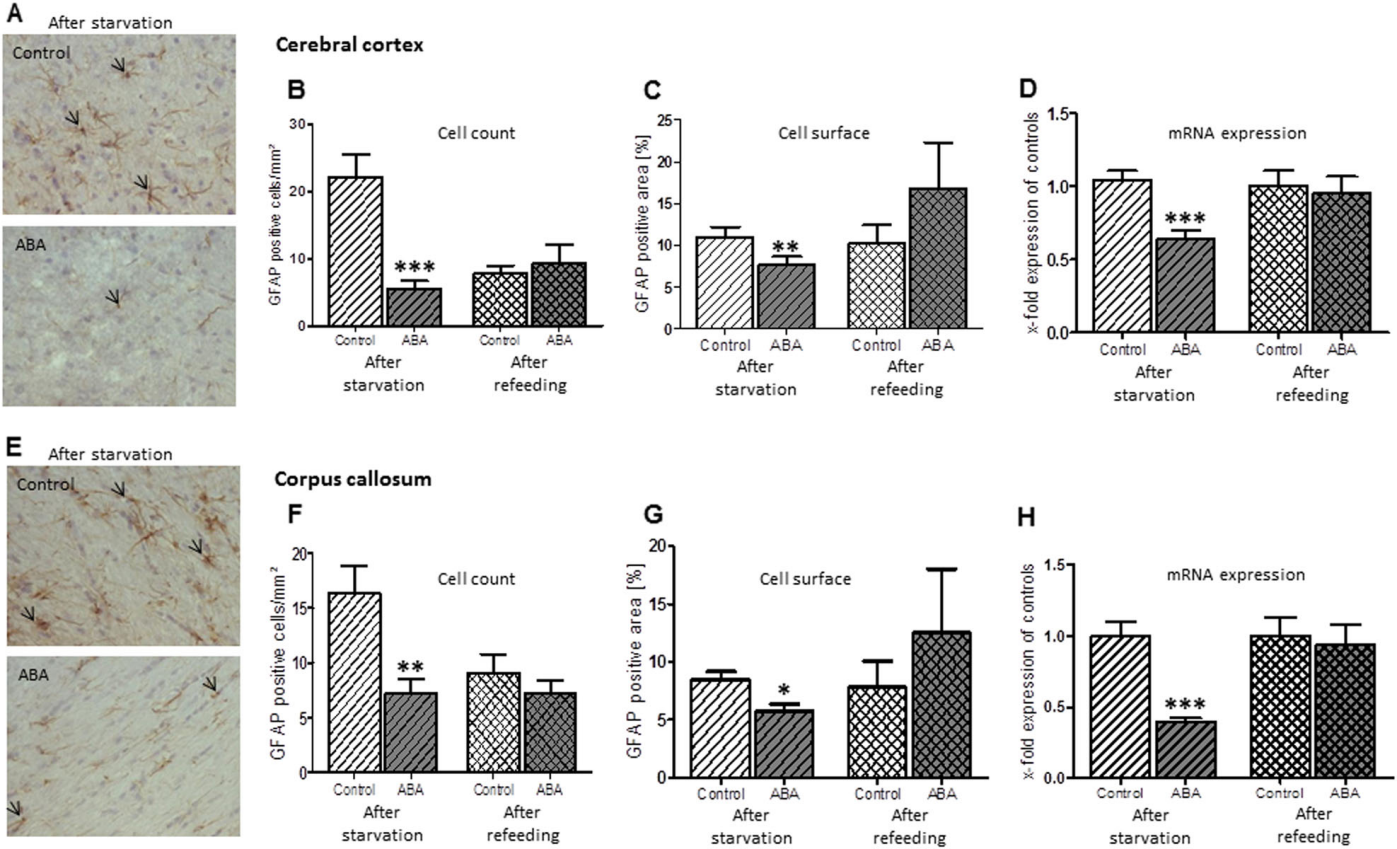
The figure shows the mean number of astrocytes, mean GFAP-positive cell surface area and mean mRNA expression of GFAP with respective SDs after starvation and refeeding in the cerebral cortex (**a**) and corpus callosum (**e**). In the cerebral cortex and corpus callosum, the number of astrocytes was significantly reduced compared to controls following starvation, and this reduction was recovered after refeeding (**b**, **f**, respectively). Similarly, the total cell surface areas in both regions were significantly reduced following starvation but not refeeding (**c**, **f**). Furthermore, the ABA group showed a significant reduction in GFAP mRNA expression in both regions following starvation, while no alterations occurred after refeeding (**d**, **h**). *p ≤ 0.05; **p ≤ 0.01; ***p ≤ 0.001, two-sided Student’s *t*-test

The mean number of GFAP-stained astrocytes in the cerebral cortex in the ABA rats was significantly reduced by 75% compared to the control group (t(df) = 22, t = 4.75, Cohen’s d = 1.94, *p* ≤ 0.001, Fig. 4, Supplementary Table 2). Additionally, in the corpus callosum, the number of cells was also diminished in the ABA animals by 56% (t(df) = 22, t = 3.24, Cohen’s d = 1.32, *p* ≤ 0.01). There were no statistically significant differences in the mean number of Map2-stained neurons (cortex: after starvation: *p* = 0.97, after refeeding: *p* = 0.38). After refeeding, the number of astrocytes in the cerebral cortex and corpus callosum of ABA_R was not significantly altered (cortex: *p* = 0.72, corpus callosum: *p* = 0.39).

The mean cell surface of the astrocytes was reduced after chronic starvation (cortex: t(df) = 22, t = 4.22, Cohen’s d = 1.72, *p* ≤ 0.001, corpus callosum: t(df) = 22, t = 3.14, Cohen’s d = 1.28, *p* ≤ 0.01, Fig. 4), but there was no statistically significant difference after refeeding (cortex: *p* = 0.18, corpus callosum: *p* = 0.11).

To substantiate the finding of the astrocyte reduction, we analyzed GFAP mRNA expression. The mean level of GFAP mRNA in the cerebral cortex and corpus callosum of the ABA rats was significantly decreased by 39% and 53%, respectively (cortex: t(df) = 22, t = 4.59, Cohen’s d = 1.87, *p* ≤ 0.001, corpus callosum: t(df) = 22, t = 4.68, Cohen’s d = 1.91, *p* ≤ 0.001, Fig. 4). After refeeding, no difference persisted (cortex: *p* = 0.77, corpus callosum: *p* = 0.69).

**Fig. 5:**
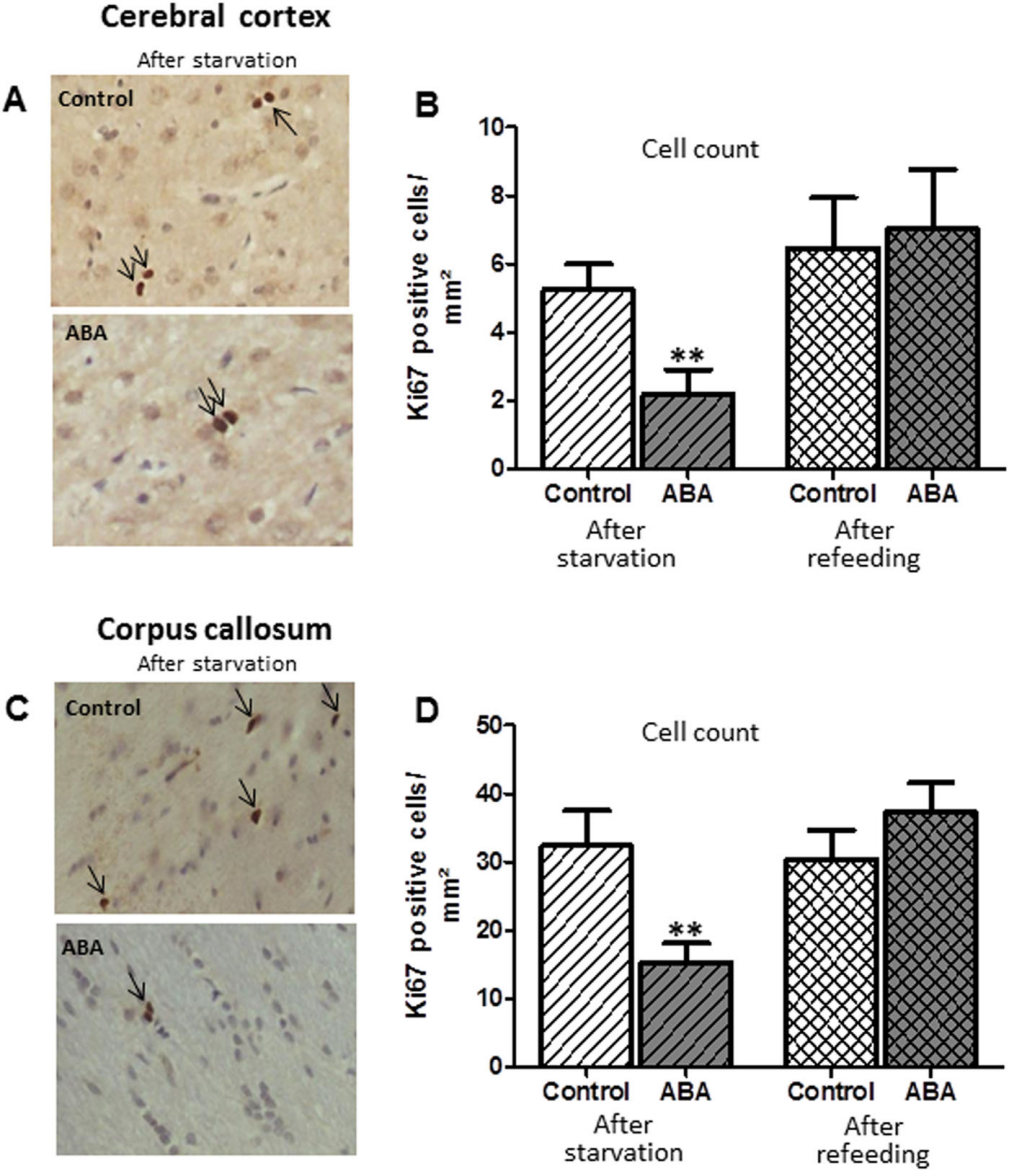
The number of Ki67-positive cells (marker for cell proliferation) was reduced in the cerebral cortex (**a**, **b**) and corpus callosum (**c**, **d**) after starvation and normalized after refeeding. **p ≤ 0.01, two-sided Student’s *t*-test

The mean number of proliferating cells, as measured with the marker Ki67, in the cortex and corpus callosum of the ABA rats was significantly reduced by 34 and 51%, respectively, compared to the controls (cortex: t(df) = 22, t = 2.95, Cohen’s d = −1.2, *p* ≤ 0.01, corpus callosum: t(df) = 22, t = 2.89, Cohen’s d = 1.18, *p* ≤ 0.01, Fig. 5). No change was noted after refeeding in this analysis. Furthermore, we measured the number of apoptotic cells with the marker Casp3 (Supplementary Fig. 2). In the cortex, the mean number of Casp3-positive cells in the ABA animals was reduced by 71%, while in the corpus callosum, no significant alteration occurred between the two groups (cortex: t(df) = 22, t = 2.87, Cohen’s d = −1.17, *p* ≤ 0.01, corpus callosum: *p* = 0.22). All raw data and statistics can be found in Supplementary Table 2.

## Discussion

The primary goal of our study was to analyze the mechanisms underlying brain volume reduction in anorexia nervosa. The present investigation explored whether the brain volume reduction in AN compared to healthy controls is associated with astrocyte loss, as proposed by our exploratory study, and this hypothesis was confirmed. The brain volume in the ABA animals did not increase as appropriate for adolescent age and was significantly reduced compared to controls in our MRI studies. We found a loss of GFAP-positive astrocytes after approximately 3 weeks of starvation compared to the control animals. We then analyzed whether the starvation-induced volumetric and cellular changes in the brain were reversible upon refeeding. This was mostly confirmed; the total volume of the cortex appeared to be reinstated, and only the white matter in the corpus callosum was still reduced. Furthermore, the cell surface of GFAP-positive cells and GFAP mRNA expression normalized after refeeding. Finally, we investigated whether the cell reduction was derived from reduced proliferation or increased apoptosis. Proliferation was reduced to less than 50%, while there was no evidence of increased apoptosis. Reversible astrocyte loss underlying the brain changes in the ABA model is a very important and new finding. If these findings are also be the case in AN patients, this knowledge could help us to understand the pathophysiological mechanisms of AN with astrocytic and ensuing neuronal dysfunction.

### Astrocyte cell reduction

The results fit well with those of the proof-of-concept study, which pointed to an astrocyte reduction in ABA rats^26^ and with those of studies by Reyes-Haro et al., who used the dehydration rat model^32^. The use of dehydration could potentially have inhibited the latter from also measuring brain volumes; thus, our findings extended their results to show a direct association between brain volume deficits and cellular changes. The astrocytic mRNA analysis further strengthened our findings by including a different method that yielded similar reductions. The cell reduction seemed to be specific to astrocytes, as neurons and oligodendrocytes were not reduced in terms of size or number in our pilot study^26^. There is a growing body of research on the multiple roles of astrocytes in the brain, as astrocytes appear to have been underestimated as structural components. The functions of astrocytes indeed include an active role in the blood-brain barrier, transporting nutrients and messengers from the bloodstream to the brain^33^. However, astrocytic functions have now been shown to include complex regulatory mechanisms, such as neurotransmitter reuptake and synapse formation, both directly and indirectly influencing learning and brain plasticity^34–36^. Astrocytes can form a syncytium using calcium influx to communicate directly between cells; they have receptors for various neurotransmitters and can react by emitting specific gliotransmitters^33,37–39^. Astrocytes have been previously implicated in complex phenomena such as sleep and mood: they appear to be involved in regulating the brain rhythms that mediate sleep function. Astrocytes are reduced in depressed patients, and impairing astrocytes in the frontal cortex leads to depressive-like symptoms in animal models^40^. Their reduction in AN could explain the neural dysfunctions and equivalent symptoms of sleep disturbance, depression and impaired learning commonly found in patients with AN^2,41,42^. By impairing proper synapse formation and plasticity, astrocytes could be responsible for the decreased cognitive flexibility and increased attention to detail that are often neuropsychological hallmarks of patients with AN. Together with the lack of insight and the low motivation to change, this could explain the difficulties encountered in the cognitive processes implicated in the psychotherapy of patients with AN during acute starvation. During long-term chronic starvation, astrocyte loss and the metabolic and functional consequences of this loss could be in part responsible for the behavioral neurodegenerative symptoms^43^. Morphological studies have also shown that the brain volume loss in these patients worsens the longer the illness lasts^15,44^.

### Reversibility of volume and cell reduction after weight gain

The results of this study showed, for the first time, the almost complete reversibility of brain volume reduction and astrocyte reduction upon refeeding. The former finding fits well with human MRI studies that have shown largely reversible brain volume changes upon weight gain^8,45^. Only the white matter volume seemed to remain reduced in our study. This could have been an effect of the relatively short time span, with more time being required for complete restauration. Alternatively, it could point to some sort of longer-lasting scarring in the still developing brain. Previous meta-analyses seemed to suggest different mechanisms underlying white versus gray matter volume rehabilitation^7,8^. In patients, gray matter loss has also been found to be strongly coupled with a low body mass index. Thus, this may be a state marker for starvation, whereas white matter had a stronger predictive component towards the outcome after one year^12^. In addition, diffusion-weighted images have shown white matter tracts to be altered not only during acute starvation^16,46^ but also partly after weight rehabilitation^47^ (however see Travis et al.^48^). Both astrocyte count and size seemed to normalize after refeeding in our study. This was a hopeful result, as it showed the reversibility of the astrocyte loss after timely weight gain. Translated to patient care, this argues for the need for a strong focus on nutritional rehabilitation and weight gain at the beginning of therapy. More studies using longer starvation periods are needed to examine whether the astrocyte cell reductions remain reversible after even longer time courses.

### Reduced cell neogenesis

We found a significant reduction in cell neogenesis, as evidenced by the decrease in Ki67-marked proliferative cells, whereas cell apoptosis was not increased. This finding corresponds with that of a study by the group of Barbarich-Marsteller (2013). They found a reduction in proliferation in regions with known glial but very little neuronal cell neogenesis, such as the non-dentate gyrus hippocampus and the corpus callosum, under acute ABA conditions. In their study, the cerebral cortex was not analyzed, and they used Ki67 staining and BrdU for proliferation measurement^30,49^. Therefore, the brain volume reduction seen in ABA could be due to the reduced proliferation in the previously mentioned brain regions. Thus, it could be that the astrocyte proliferation in corpus callosum and cortex was reduced because there is only very limited neurogenesis in these regions. This finding would fit well with the catabolic state that occurs during starvation, where the energy to build new cells would be scarce rather than favorable to more rapid apoptosis.

### Limitations

The presented results refer to animal data only, and special care must be taken when translating animal research to human patients. However, the ABA model is the most widely used AN animal model with good congruence in all somatic symptoms of patients with AN (weight loss, endocrinological changes, brain volume loss, etc.), so the translational significance appears to be high^50,51^. Nevertheless, human post-mortem studies of patients with AN are definitely needed. Brain atrophy in the ABA animals could have slightly influenced the cell count (reference trap in stereology^52^). Because of this volume reduction, the cell count per volume would be expected to be approximately 6–9% too high in ABA animals, so the true GFAP-positive astrocyte reduction could be expected to be even slightly more pronounced. Furthermore, our astrocyte marker GFAP is a marker for adult, differentiated astrocytes;^53^ therefore, we do not know whether the astrocyte loss shown here extends to immature, undifferentiated astrocytes. Further, also neuronal progenitor cells are stained with this marker, but these cells are only prominent in regions like the subventricular zone of the lateral ventricle and in the dentate gyrus of the hippocampus^54^. Lastly, we only analyzed brain structure and the underlying cellular base but did not study the functional consequences of astrocyte loss and how it influences neuronal function and behavior.

### Consequences

As stated above, a dramatic loss of astrocytes in the ABA model does not bode well for the (developing) brain and behavior of patients. If brain volume reduction and astrocyte loss are reversible after a relatively short period of starvation in ABA, rapid weight gain needs to be a priority in the care of patients with AN. Furthermore, the time-scale of astrocyte loss and regeneration as well as the degree to which astrocyte functionality is also impaired, along with the cell count reduction, needs to be further researched. The questions of the specific mechanism of changes in synapse formation and modulation and whether these changes result in altered levels of neurotransmitters or gliotransmitters require future studies. Further investigation of altered astrocyte functionality could provide a better understanding of the intermediate brain alterations linking the cellular changes and psychological symptoms. Astrocyte studies are also important for starvation in general, leading to psychological symptoms^55^ and other diseases involving under or malnutrition, such as illnesses associated with cachexia (e.g., in cancer patients) and even in developing countries^56^. Consequently, astrocytes could become an entirely new focus for research not only in AN but also other psychiatric diseases that involve astrocyte cell reductions, such as depression, anxiety disorder^28^ and chronic stress^57^.

## Conclusion

Astrocytes were severely depleted in our AN animal model following starvation, which reversed upon weight restoration, possibly explaining typical neuropsychological symptoms of AN of starvation in general. Astrocytes could become an important target for further research and interventions.

## Supplementary information

Supplement

Supplementary figures
