# Supplementary material for: The reduction of astrocytes and brain volume loss in anorexia nervosa—the impact of starvation and refeeding in a rodent model
Source: Transl Psychiatry. 2019 Jun 4;9:159. doi: 10.1038/s41398-019-0493-7 (PMC6548775; doi:10.1038/s41398-019-0493-7)
Supplement: Supplementary file 1 — Supplement [file 41398_2019_493_MOESM1_ESM.pdf]

Supplementary information

Supplementary figure 1: Effect of starvation and refeeding on running wheel activity (RWA). The RWA was normalized to the habituation phase at 100% and displayed as the mean values.

Supplementary figure 2: Effect of starvation on Casp3-positive cell number. The amount of Casp3-positive cells in the cerebral cortex was reduced in the ABA group compared to control animals (A, B). No significant alteration was found in Casp3-positive cell number in the corpus callosum (C, D).

Supplementary figure 3: Illustration of chosen ROIs in corpus callosum and cerebral cortex for the measurement of cellular parameters (corpus callosum: CC1: next to the midline, CC2: under the subcingulum, CC3: lateral; cerebral cortex: CX1: retrosplenial granular cortex, CX2: primary motor cortex, CX3: primary somatosensory cortex).

Supplementary table 1: (A) The differences in mean brain volumes and standard deviations in mm^3 between the ABA and control animals before and after starvation and after refeeding, as determined with MRI. (B) Results of the corresponding linear contrasts for ABA and control animals in the linear mixed effects model with random intercept and unstructured covariance matrix expressed as degrees of freedom (t(df)), value of the test statistic (t), two-sided p-value (p-value), mean difference and 95% confidence intervals (CI).

Supplementary table 2: (A) Results of the analysis of cerebral cortex and corpus callosum volumes expressed as mm^3 with corresponding standard deviation (SD) of ABA and control animals after starvation and refeeding. (B) Astrocyte cell numbers in cells/mm^2. (C) Cell surface of GFAP-positive cells in % of the total surface. (D) mRNA expression in both analyzed regions expressed as fold change relative to the control animals (controls set to 100%). (E) Numbers of cells that were positive for Ki67, (F) Map2 and (G) Casp3 in cells/mm^2.
