# Supplementary material for: The reduction of astrocytes and brain volume loss in anorexia nervosa—the impact of starvation and refeeding in a rodent model
Source: Transl Psychiatry. 2019 Jun 4;9:159. doi: 10.1038/s41398-019-0493-7 (PMC6548775; doi:10.1038/s41398-019-0493-7)
Supplement: Supplementary file 2 — Supplementary figures [file 41398_2019_493_MOESM2_ESM.pdf]

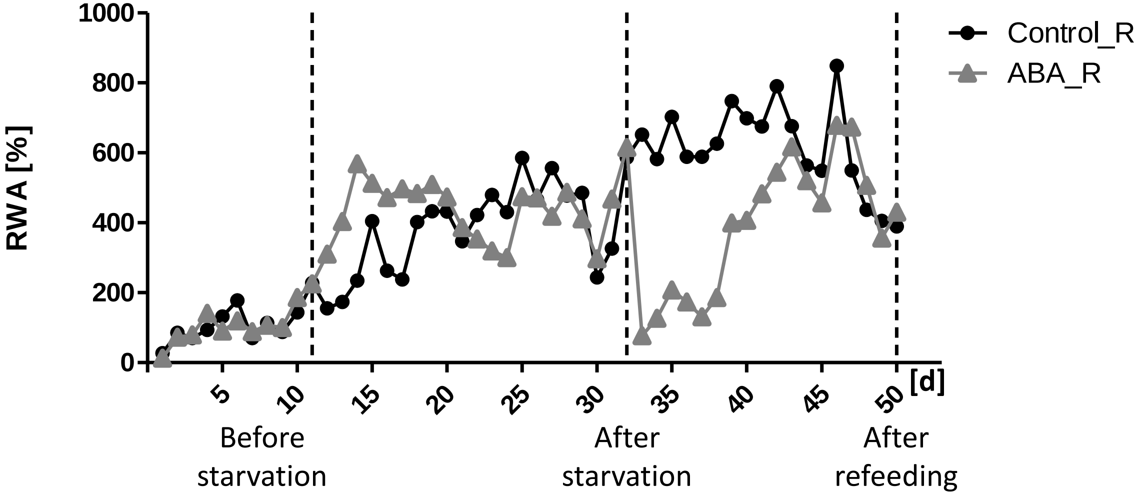
RWA [%]
1000
800
600
400
200
0
5
10
15
20
25
30
35
40
45
50
[d]
Control_R
ABA_R
Before starvation
After starvation
After refeeding

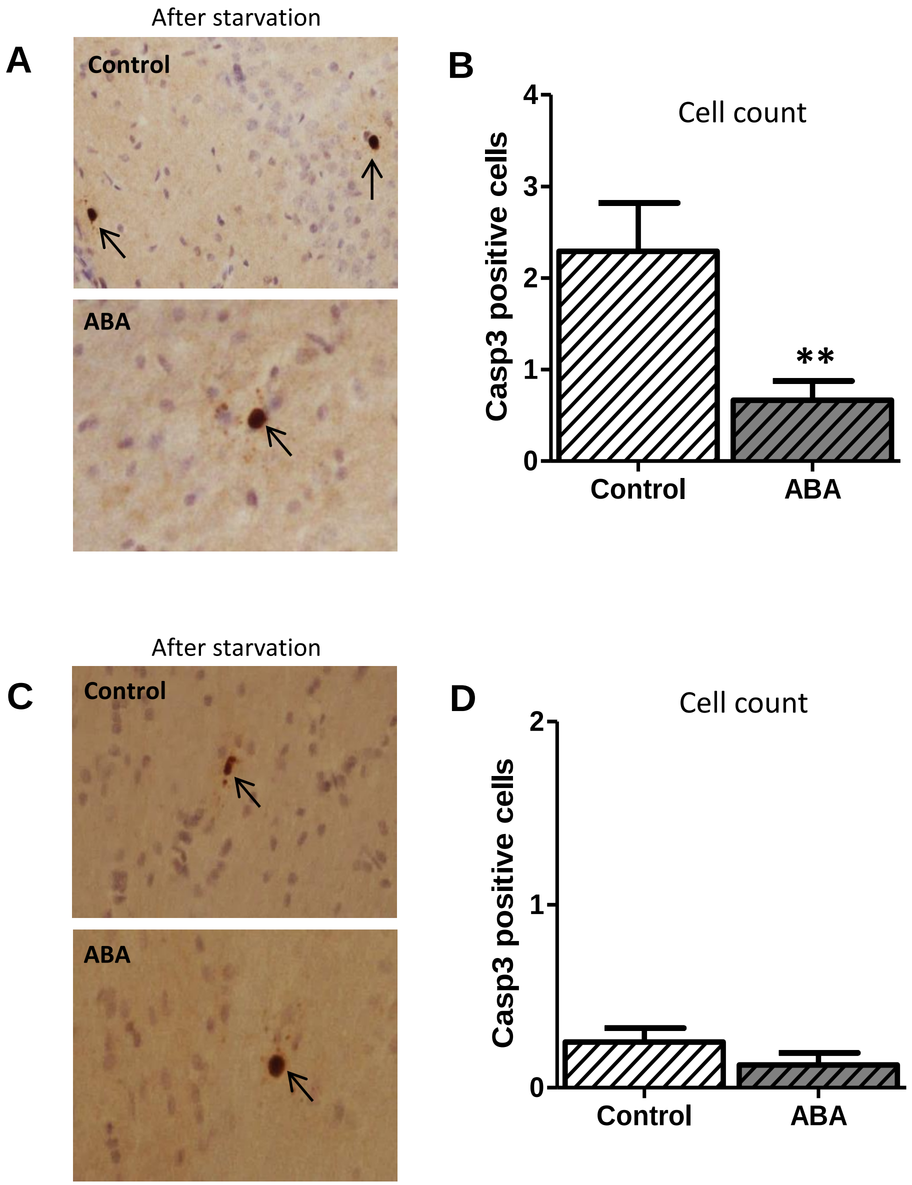

After starvation
A
Control
ABA
B
Cell count
Casp3 positive cells
4
3
2
1
0
**
Control
ABA
After starvation
C
Control
ABA
D
Cell count
Casp3 positive cells
2
1
0
Control
ABA

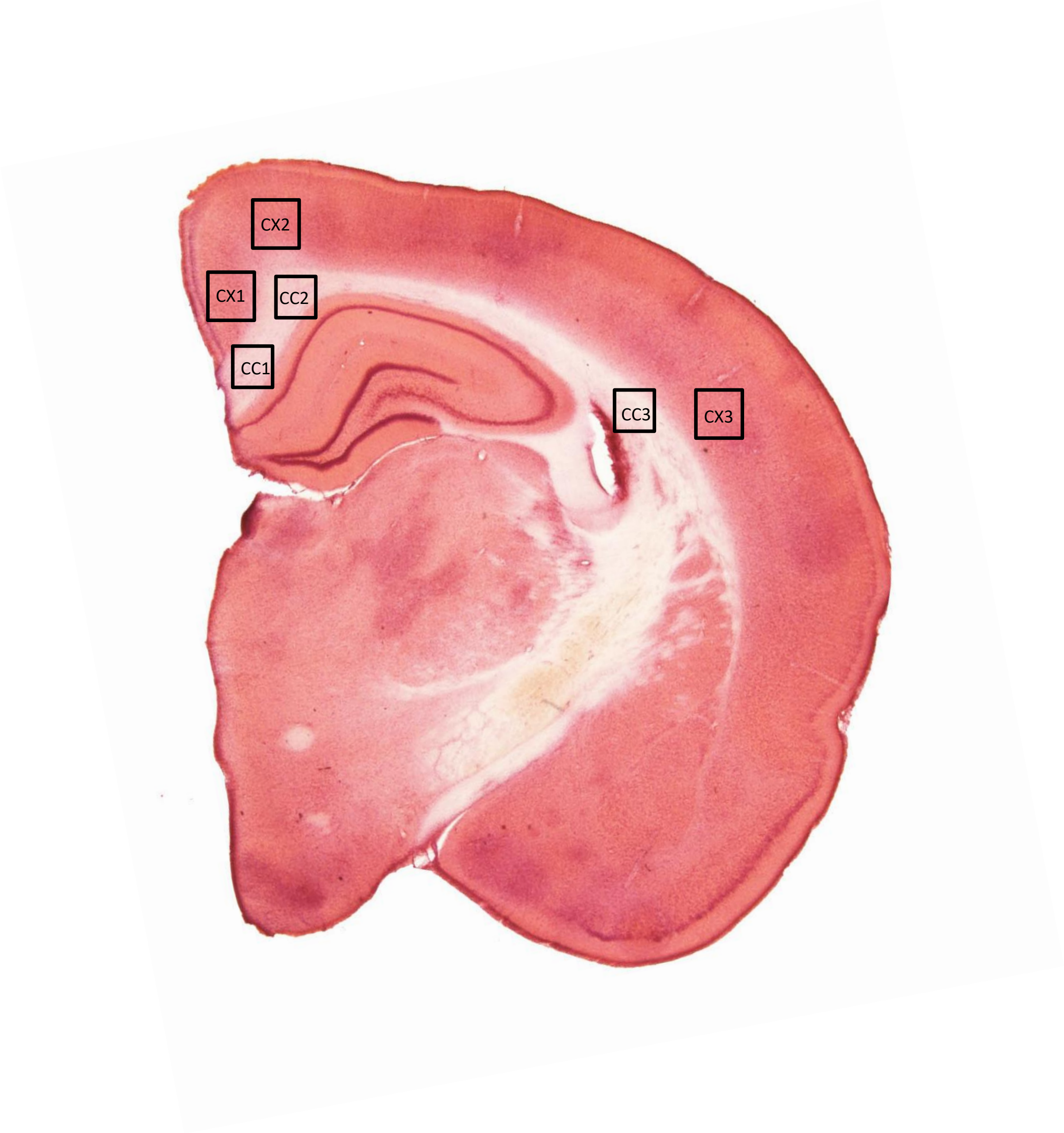
CX2
CX1
CC2
CC1
CC3
CX3

Supplementary table 1, Frintrop et al.

Table 1

A

Total brain volume, Analysis Variable : Outcome				
**Time**	**Group**	**N**	**Mean (mm³)**	**SD**
Before starvation	ABA	9	1467.33	115.47
	Control	11	1485.65	113.00
After starvation	ABA	11	1479.65	94.60
	Control	12	1581.24	114.67
After refeeding	ABA	11	1560.33	69.94
	Control	12	1588.52	57.93

B

Label	df	t	p-value	Mean difference	CI lower bound	CI upper bound
**ABA vs. control after starvation**	40.7	-2.54	0.02	-101.59	-182.53	-20.6543
**ABA vs. control after refeeding**	40.7	-0.70	0.49	-28.1841	-109.12	52.7544

Table 2

Volume Cerebral Cortex (mm^3)

	Time	Group	N	Mean	SD	p-value	t (df)	t	Cohen`s d
A	After starvation	ABA	11	188.98	5.07				
		Control	10	206.51	3.61	0.01	19	2.85	1.24
	After refeeding	ABA	10	208.45	4.56				
		Control	12	208.59	2.62	0.57	19	0.72	0.31

Volume Corpus Callosum (mm^3)

	Time	Group	N	Mean	SD	p-value	t (df)	t	Cohen`s d
	After starvation	ABA	11	22.89	0.56				
		Control	7	23.58	0.69	0.08	16	1.89	0.91
	After refeeding	ABA	11	22.34	0.68				
		Control	12	24.93	0.71	0.02	21	2.42	1.01

GFAP Cell Count (cells/mm^2)

	Variable	Time	Group	N	Mean	SD	p-value	t (df)	t	Cohen`s d
B	Cerebral Cortex	After starvation	ABA	9	5,55	3,98				
			Control	12	22,21	11,49	≤0.001	22	4.75	1.94
		After refeeding	ABA	8	9.39	7.66				
			Control	12	7.77	4.21	0.72	21	-0.36	0.15
	Corpus Callosum	After starvation	ABA	12	7.24	4.56				
			Control	12	16.36	8.62	≤0.01	22	3.24	1.32
		After refeeding	ABA	11	7.27	3.87				
			Control	12	9.09	5.77	0.39	21	0.88	-0.37

GFAP Cell surface (positive area, %)

	Variable	Time	Group	N	Mean	SD	p-value	t (df)	t	Cohen`s d
C	Cerebral Cortex	After starvation	ABA	12	7.77	0.92				
			Control	12	11.02	1.20	≤0.001	22	4.22	1.72
		After refeeding	ABA	11	16.84	5.44				
			Control	12	10.24	2.20	0.18	21	-0.79	0.33
	Corpus Callosum	After starvation	ABA	12	5.77	0.57				
			Control	12	8.50	0.67	≤0.01	22	3.14	1.28
		After refeeding	ABA	11	12.53	5.51				
			Control	12	7.79	2.32	0.11	21	-0.82	0.34

GFAP mRNA expression

	Variable	Time	Group	N	Mean	SD	p-value	t (df)	t	Cohen`s d
D	Cerebral Cortex	After starvation	ABA	12	0.64	0.22				
			Control	12	1.00	0.21	≤0.001	22	4.59	1.87
		After refeeding	ABA	10	0.95	0.36				
			Control	12	1.00	0.37	0.77	20	0.29	-0.13
	Corpus Callosum	After starvation	ABA	10	0.40	0.08				
			Control	12	1.00	0.34	≤0.001	22	4.68	1.91
		After refeeding	ABA	7	0.94	0.37				
			Control	7	1.00	0.35	0.69	15	-0.4	0.2

Ki67 Cerebral Cortex

	Variable	Time	Group	N	Mean	SD	p-value	t (df)	t	Cohen`s d
E	Cell count (cells/mm^2)	After starvation	ABA	12	2.18	2.50				
			Control	12	5.24	2.60	≤0.01	22	2.95	-1.2
		After refeeding	ABA	11	7.05	5.68				
			Control	12	6.46	5.10	0.8	21	0.26	0.11

Ki67 Corpus Callosum

	Variable	Time	Group	N	Mean	SD	p-value	t (df)	t	Cohen`s d
	Cell count (cells/mm^2)	After starvation	ABA	12	15.31	9.71				
			Control	12	32.36	17.96	≤0.01	22	2.89	1.18
		After refeeding	ABA	11	37.35	14.60				
			Control	12	30.40	13.93	0.26	21	-1.17	0.49

Map2 Cerebral Cortex

	Variable	Time	Group	N	Mean	SD	p-value	t (df)	t	Cohen`s d
F	Cell count (cells/mm^2)	After starvation	ABA	12	329.22	119.56				
			Control	12	327.53	73.27	0.97	16	0.04	0.02
		After refeeding	ABA	11	319.61	71.89				
			Control	12	295.65	56.51	0.38	21	-0.89	0.37

Casp3 Cerebral Cortex

	Variable	Time	Group	N	Mean	SD	p-value	t (df)	t	Cohen`s d
G	Cell count (cells/mm^2)	After starvation	ABA	12	0.67	0.74				
			Control	12	2.29	1.83	≤0.01	22	2.87	-1.17

Casp3 Corpus Callosum

	Variable	Time	Group	N	Mean	SD	p-value	t (df)	t	Cohen`s d
	Cell count (cells/mm^2)	After starvation	ABA	12	0.13	0.23				
			Control	12	0.25	0.26	0.22	22	1.25	-0.51
